# Structural neuroimaging markers of normal pressure hydrocephalus versus Alzheimer’s dementia and Parkinson’s disease, and hydrocephalus versus atrophy in chronic TBI—a narrative review

**DOI:** 10.3389/fneur.2024.1347200

**Published:** 2024-03-21

**Authors:** Sharada Kadaba Sridhar, Jen Dysterheft Robb, Rishabh Gupta, Scarlett Cheong, Rui Kuang, Uzma Samadani

**Affiliations:** ^1^Department of Bioinformatics and Computational Biology, University of Minnesota, Minneapolis, MN, United States; ^2^Neurotrauma Research Lab, Center for Veterans Research and Education, Minneapolis, MN, United States; ^3^University of Minnesota Twin Cities Medical School, Minneapolis, MN, United States; ^4^Department of Computer Science and Engineering, University of Minnesota, Minneapolis, MN, United States; ^5^Division of Neurosurgery, Department of Surgery, Minneapolis Veterans Affairs Health Care System, Minneapolis, MN, United States

**Keywords:** normal pressure hydrocephalus, Alzheimer’s dementia, Parkinson’s disease, chronic traumatic brain injury, structural imaging, computational methods

## Abstract

**Introduction:**

Normal Pressure Hydrocephalus (NPH) is a prominent type of reversible dementia that may be treated with shunt surgery, and it is crucial to differentiate it from irreversible degeneration caused by its symptomatic mimics like Alzheimer’s Dementia (AD) and Parkinson’s Disease (PD). Similarly, it is important to distinguish between (normal pressure) hydrocephalus and irreversible atrophy/degeneration which are among the chronic effects of Traumatic Brain Injury (cTBI), as the former may be reversed through shunt placement. The purpose of this review is to elucidate the structural imaging markers which may be foundational to the development of accurate, noninvasive, and accessible solutions to this problem.

**Methods:**

By searching the PubMed database for keywords related to NPH, AD, PD, and cTBI, we reviewed studies that examined the (1) distinct neuroanatomical markers of degeneration in NPH versus AD and PD, and atrophy versus hydrocephalus in cTBI and (2) computational methods for their (semi-) automatic assessment on Computed Tomography (CT) and Magnetic Resonance Imaging (MRI) scans.

**Results:**

Structural markers of NPH and those that can distinguish it from AD have been well studied, but only a few studies have explored its structural distinction between PD. The structural implications of cTBI over time have been studied. But neuroanatomical markers that can predict shunt response in patients with either symptomatic idiopathic NPH or post-traumatic hydrocephalus have not been reliably established. MRI-based markers dominate this field of investigation as compared to CT, which is also reflected in the disproportionate number of MRI-based computational methods for their automatic assessment.

**Conclusion:**

Along with an up-to-date literature review on the structural neurodegeneration due to NPH versus AD/PD, and hydrocephalus versus atrophy in cTBI, this article sheds light on the potential of structural imaging markers as (differential) diagnostic aids for the timely recognition of patients with reversible (normal pressure) hydrocephalus, and opportunities to develop computational tools for their objective assessment.

## 1 Introduction

Normal pressure hydrocephalus (NPH), is a prominent type of dementia that is often reversible via ventricular shunt surgery, with earlier intervention leading to better outcomes (1). A notable discrepancy between the incidence (of patients who had surgical intervention) (2–4) and prevalence rates (5–7) suggests its under-recognition. This is substantiated by an estimate from the Hydrocephalus Association that 80% of patients with NPH remain unrecognized with most frequent misdiagnoses being Alzheimer’s Dementia (AD) or Parkinson’s Disease (PD) (8), which themselves significantly contribute to the global burden of neurological disorders (9). About half of the cases of NPH are estimated to be idiopathic, and the other half secondary to traumatic brain injury (TBI), tumor, meningitis, or infections (10, 11). Adding complexity to its recognition may be the fact that in the secondary NPH group, while tumors or infection linked to the disease can be detected definitively, a TBI-related origin may not be accurately identified due to incomplete medical history. Therefore, the accurate detection and treatment of NPH may inevitably depend on discerning it from irreversible atrophic pathologies like AD, PD, and post-traumatic degeneration.

In terms of structural neurodegeneration, hydrocephalic ventriculomegaly is the key marker of NPH (12). Idiopathic NPH and NPH secondary to TBI, may differ in their etiology, but remarkable improvement in symptoms of patients with both conditions have been demonstrated after ventricular shunting (1, 13). But because atrophy is a chronic effect of TBI which may cause secondary ventriculomegaly, and has been negatively correlated with shunt outcome, accurately discerning it from hydrocephalic ventriculomegaly on structural imaging (14) is paramount for shunt-surgery decision making. The structural degeneration in AD (15) and PD (16) is also characterized by atrophy. Both atrophy and hydrocephalus can give an appearance of ventricular enlargement, which may set a precedent for misdiagnosis if not examined carefully.

In addition to similar structural neurodegeneration, the cognitive and functional deficits such as dementia and gait impairment caused by these diseases often overlap, rendering misdiagnoses distressingly common. The stretching of the corticospinal tract (CST) in the corona radiata which conducts signal to the legs is thought to produce gait disturbance, a manifestation in most NPH cases, while radial shearing force exerted by enlarging ventricles leads to dementia (17). Loss of structural integrity leads to impairment of cognitive and executive function in AD which may affect gait due to divided attention (18). PD which is thought to arise in the substantia nigra and basal ganglia, is characterized by its motor symptoms including gait impairment (19). Its degenerative impact extends well into the cerebral cortex as atrophy leading to cognitive deficits (20). Chronic TBI (cTBI) also leads to cognitive and gait impairment through its degenerative effects (21, 22).

The neurostructural damage resulting from NPH manifests as distinct imaging markers capturable on Computed Tomography (CT) and Magnetic Resonance Imaging (MRI). CT-based markers have been included as a supporting factor in the diagnostic guidelines for NPH and acute TBI. However, they have not gained prominence relative to neurological, clinical, cerebrospinal fluid (CSF) and blood biomarkers, advanced MRI, and functional imaging, especially in AD, PD, and cTBI. Structural imaging markers may offer a noninvasive solution not only for the accurate detection of NPH, but also for its distinction from irreversible neurodegeneration in AD and PD; and distinguishing between atrophy and hydrocephalus in cTBI. There have been a handful of reviews that have elucidated imaging markers of NPH. Pyrgelis et al. (23) provided a review of functional and structural imaging markers for NPH and highlighted a limited number of them which distinguish it from AD and PD, and Yin et al. (24) presented a mini-review of NPH-specific features. But there have been no focused attempts to highlight the structural imaging markers of NPH along with those that can differentiate it from its mimics AD and PD and shed light on the interplay of atrophy and hydrocephalus in cTBI. This review also comments on the adoption of computational techniques for the objective assessment of these markers and highlights areas where further research is needed.

## 2 Methodology

We searched PubMed for the keyword “normal pressure hydrocephalus” in combination with the keywords “Alzheimer” and “parkinson.” We also searched for “hydrocephalus” and “atrophy,” in combination with “traumatic brain injury.” Studies that used only structural neuroimaging modalities of (T1/T2/DTI) MRI and CT, and those that studied humans aged 19 years and above were retained. From the resultant set of articles, we present this review of structural imaging markers for NPH. For AD and PD, we provide a brief background of their burden, pathology, structural degenerative markers that differentiate NPH from them. The review of cTBI, and consequent atrophy and hydrocephalus is structured similarly. Articles where (semi) automatic image processing and machine (deep) learning have been applied to assess these features in MRI and CT modalities are also discussed. The key abbreviations and structural imaging marker definitions are in Tables 1, 2 respectively.

**Table 1:** Key abbreviations.

Abbreviation	Description
CSF, WM, GM, ICS, TIV	Cerebrospinal Fluid, White Matter, Gray Matter, Intracranial Space, Total Intracranial Volume
SF, HC, Prtl-C	Sylvian Fissure, High Convexity, Parietal Convexity
ML, SS	Midline, Subarachnoid Space
LV, 3 V, 4 V, TH, FH, *VS*	Lateral Ventricles, Third Ventricle, Fourth Ventricle, Temporal Horn, Frontal Horn, Ventricular Space
AC, PC	Anterior Commissure, Posterior Commissure
DESH	Disproportionately Enlarged Subarachnoid Space Hydrocephalus
PV, CM	Periventricular, Cella Media
CC, CST	Corpus Callosum, Corticospinal tract
lt, bl, mdl, l, r, AP, IS	lateral, bilateral, medial, left, right, anterior–posterior, inferior–superior
CT, HU	Computed Tomography, Hounsfield Unit
MRI (Magnetic Resonance Imaging)	T1/T2 weighted MRI: T1 enhances signal from fatty tissue and suppresses that from water, whereas T2 enhances the signal from water content.
	Fluid attenuated inversion recovery (FLAIR): Used to null out signal from fluid and fat tissues, particularly useful to examine the periventricular space
Diffusion Tensor Imaging (DTI) MRI	Used to capture microstructural integrity based on the underlying physiology of water diffusion in tissues
	Mean Diffusivity (MD): Captures rotationally invariant diffusivity
	Radial Diffusivity (RD): Captures perpendicular diffusivity, more specific to demyelination
	Axial Diffusivity: Captures parallel diffusivity, more specific to axonal degeneration
	Fractional Anisotropy (FA): Measures anisotropy which is affected by WM damage

**Table 2:** Structural imaging markers and definitions.

Marker	Definition
CA	Callosal Angle. Angle between lateral ventricles as viewed on the coronal plane perpendicular to the AC-PC line at the level of the PC (25)
simpCA	Simplified *CA.* Angle between the LV on the coronal plane perpendicular to the AC-PC line at the level of the midpoint of the CC (26)
ACA	Anterior *CA.* Angle between lateral ventricles as viewed on the coronal plane perpendicular to the AC-PC line at the level of the AC (27)
EI-x	Evans index. Ratio of the maximum width of the frontal horns of the LV (Frontal Horn Diameter (FHD)) and the brain width/inner cranial width as measured on consecutive axial planes parallel to the AC-PC line (25)
mFHI	Ratio of maximal frontal horn width to the bicortical width on the same plane (28)
EI-y	Evans-y index. Ratio of the maximum length of the frontal horns of the LV from the foramen of Monro in the AP direction and the brain length/inner cranial length (29)
EI-z	Evans-z index. Ratio of the maximum height of the frontal horns of the LV from the foramen of Monro in the IS direction and the brain height/inner cranial height (29)
SA	Splenial Angle. Angle subtended by the forceps major of the CC at the midline on the first axial plane with the complete body of the CC visible while moving in the IS direction (30).
CH	Callosal Height. Maximum perpendicular distance between the inferior level of the CC and the line connecting the anterior and posterior extremities of the CC assessed on the midsagittal plane (31)
mCMI	Modified Cella Media Index. Ratio of the maximum cella media width as seen on axial planes normalized by the brain width on the same plane (32).
CMW	Maximum Vertical Width of the Cella Media of the LV. Maximum vertical width of the cella media as seen on the coronal plane at the level of the PC (31)
mTHW	Mean Temporal Horn Width, as seen on axial views (33)
THW	Maximum Vertical Width of the Temporal Horn of the LV, as seen on the coronal plane at the level of the PC (31)
CTR	Ratio of the CMW and TMW (31)
DESH	Disproportionately Enlarged Subarachnoid Space Hydrocephalus. Captures the relationship between high convexity tightness, enlarged sylvian fissure, and ventriculomegaly as a descriptive indicator (34)
SILVER index	The ratio of the SF area to the subarachnoid space area at the vertex measured on a coronal plane (perpendicular to transverse planes parallel to the frontal fossa) at the level of the foramen of Monro (35)
ALVI	Anteroposterior diameter of the lateral ventricle index. Defined as the AP diameter of the LV on the first axial plane with the CM completely visible normalized by the inner brain length along the falx on the same plane (36)
iNPH Radscale	Visual grading, that is a combination of the EI-x, CA, mean width of the TH, narrow HC sulci, dilated SF, focally dilated sulci, and PV hypodensities. Higher values indicate a more severe NPH pathology (37)
BVR	Brain to ventricle ratio. Ratio of the SVW and CMW (31). Ratio of the maximum brain width divided by the maximum LV width assessed on coronal sections at the AC and PC levels (38)
CCD	Callosal-commissural distance. Distance between the inferior level of the CC and the PC assessed on the midsagittal plane, on a line parallel to posterior brain stem margin (31)
CVD	Callosal-ventricular distance. Distance between the inferior level of the CC and the line connecting the roofs of the left and right LVs assessed on the coronal plane at the level of the PC (31)
SVW	Maximum Vertical Width of the Supratentorial Brain. Maximum vertical width of the brain in the supratentorial space assessed on the coronal plane at the level of the PC (31)
BCVI	Bicaudate cerebroventricular index. Ratio of the frontal horns of the LV to the bihemispheric width (39)
Hemispheric CVI	Ratio of each of the frontal horns of the LV to the hemisphere specific width (39)

## 3 Normal pressure hydrocephalus

Hakim and Adams, first explained the mechanism of NPH using Pascal’s law. Presently, at least 700,000 older adults in the US are estimated to be afflicted by the condition (40). Excessive buildup of CSF in the ventricles leads to their enlargement and impingement on the surrounding brain tissue. And due to the compliance of the surrounding brain tissue, intracranial pressure (ICP), remains “normal” (12). Recent studies suggest that the pathogenesis of NPH is complex and is potentially related to CSF dynamics, CSF – interstitial fluid exchange, cortical subarachnoid space (SS) morphology, and venous congestion (41). It is clinically characterized by the Hakim triad which is a combination of gait impairment, urinary incontinence, and cognitive impairment.

The American-European diagnostic guidelines classify NPH into possible and probable subgroups based on clinical evaluation for the Hakim triad, an invasive CSF tap-test with opening CSF pressure of 5–18 mm Hg, medical history, and radiographic assessment of lateral ventricle (LV) enlargement (42). Enlarged temporal horns (TH) and periventricular intensity changes unattributable to ischemia or demyelination are also considered. While they do not endorse a classification of patients with definite NPH based on positive shunt response, the Japanese guidelines take that approach, and include evaluation of sulcal tightness at the high-convexity (HC) of the brain and dilation at the sylvian fissures (SF) (43). Age at onset and presence of other comorbidities are also considered in both guidelines (44). Irrespective of different approaches to classification, a vast majority of patients saw favorable outcomes following shunt surgery (45) and early surgical intervention was found to significantly affect functional outcomes (46). And despite reports of complications (47), advances in surgical procedures guided by computer aided neuronavigation, and infection reduction strategies suggest a promising future for complete reversal of symptoms in NPH (48).

### 3.1 Structural imaging markers of NPH

Radiological markers are significantly factored in the clinical diagnosis of NPH. Diagnostic standard measures of ventriculomegaly in NPH diagnosis include the callosal angle (CA), Evans-x index (EI-x), and the Disproportionately Enlarged Subarachnoid Space Hydrocephalus (DESH) (42, 43). Several other structural markers describing the effects of NPH on the ventricular and CSF spaces, white matter (WM), and gray matter (GM) structures have been described over the years. The prominent features described in this section are summarized in Table 3.

**Table 3:** Structural imaging markers of NPH.

Disease and comparison groups	Structural marker	References	Assessment	Imaging modality
14 probable NPH, 5 definite NPH, 12 NC	CSF volume at the HC, ML: NPH < NC	(49)	Manual	T1 – MRI
24 probable NPH, 24 tap-negative NPH, 23 NC	EI-z: NPH > NC; CSF volume at the Prtl-C, Ratio of CSF volume in upper and lower SS (split by a transverse plane parallel to the AC-PC line, at the level of junction of the straight sinus and vein of Galen): NPH < NC	(29)	Semi-Automatic	T2 – MRI
29 possible NPH (22 probable, 25 definite), 26 NC	SILVER index: NPH > NC	(35)	Manual	CT
75 definite NPH, 55 NC	iNPH Radscale: NPH > NC	(50)	Manual	CT
23 definite NPH, 62 NC	ALVI: NPH > NC	(36)	Manual	CT
140 NPH (33 possible, 53 probable, 54 definite), 52 NC	Ratio of GM (at the thalamus) and WM (anterior bilateral PV space) density (HU): NPH > NC	(51)	Manual	CT
21 definite NPH, 20 NC	CSF volume, GM volume in the precuneus, paracentral lobules (bl), supplementary motor area (bl), left cerebral hemisphere (mdl), cingulate (mdl), paracingulate gyri: NPH > NC; WM volume, GM volume in the temporal lobes (bl), thalamus (bl), hippocampus (bl), insula (bl), amygdala (l), lenticular nucleus (r), putamen (r), and cerebellum: NPH < NC	(52)	Semi-Automatic	T1 and T2 – MRI
35 possible NPH (89% probable), 45 NC	CA, simpCA, BVR: NPH < NC; EI-z, EI-x, DESH, FHD, FHVD, SVW, CVD, CCD, CH, CMW, THW: NPH > NC	(31)	Manual	T1 – MRI
16 possible NPH, 14 NC	Volume of hippocampus, normalized by the intracranial supratentorial volume: NPH < NC	(53)	Manual	Inversion recovery, T1 – MRI
10 possible NPH, 21 headache controls	Posterior part of the cingulate gyrus tighter/narrower than anterior part (Cingulate Sulcus Sign): NPH > headache controls; Concavity of the upper midbrain profile (Upper Midbrain Profile Sign): NPH > headache controls	(54)	Manual	T1, T2, FLAIR MRI
14 NPH, 8 NC	Volumes of the caudate, putamen, thalamus, nucleus accumbens, hippocampus, and pallidum: NPH < NC	(55)	Semi-Automatic	T1 – MRI

NC, Normal Controls.

As CSF spaces are the most distinctly deformed anatomy in NPH, features describing its volume and morphology have been extensively studied and applied in the detection of NPH. A 2007 study of 14 patients with probable NPH (3.5% with shunt response) patients on MRI scans indicated that visually apparent narrowing of CSF spaces at the HC and midline (ML) accurately separated them from age-matched controls (49). Another study of 24 patients with probable NPH who had functional improvement after a CSF-tap test (tap positive), 24 tap negative patients, and 23 age matched controls using on (T2) MRI scans showed that 3 newly proposed metrics – CSF volume at the parietal convexity (Prtl-C), the Evans-z index (EI-z), and upper to lower SS ratio were optimal diagnostic indices of iNPH as they had the highest area under the curve of receiver operating characteristics (AUCROC) in distinguishing between tap-positive and tap-negative patients. Absolute and normalized (by intracranial volume) volumes of the total ventricle and bilateral ventricle spaces, the Evans-y index (EI-y), EI-x, maximum ventricle to brain lengths (in the 3 orthogonal directions), CA, CSF volumes of the SS at the frontal and parietal convexity, SF and basal cistern, and posterior fossa were also among the evaluated features (29).

Changin et al. proposed a simplified CA (simpCA) measure to overcome the need for extensive image processing to obtain the CA and showed that it was significantly lowered in NPH patients as seen on MRI sequences (27). To mitigate nonuniformity and examination of multiple slices required to measure the EI-x, another quantitative measure called the anteroposterior diameter of the lateral ventricle index (ALVI) on a fixed a standard plane was demonstrated to be better correlated with ventricular volume as compared to the EI-x, and a threshold of 0.5 could detect patients with ventriculomegaly in definite NPH when compared to healthy controls on CT (36). A comprehensive study of interpretable measures of the CSF spaces on MRI evaluated the frontal horn diameter (FHD), DESH, CA, simpCA, maximum vertical width of the supraventricular brain (SVW), cella media width (CMW), temporal horn width (THW), frontal horn vertical diameter (FHVD), callosal ventricular distance (CVD), callosal commissural distance (CCD), callosal height (CH), EI-x, EI-z, cella media to temporal horn ratio (CTR: CMW/THW), and brain to ventricle ratio (BVR: SVW/CMW), showed that all features except the CTR were able capture significant differences between probable NPH and normal controls. However, the capacity of these features in predicting shunt response were not evaluated in the study (31).

Structural imaging markers that predict shunt response in NPH have not been reliably established. A larger study of probable NPH (*n* = 229) patients contrasted with a non-NPH group, manually examined volumes of the LVs, basal cisterns, and SS superior to and at the level of the SF. They also manually evaluated DESH, focally dilated sulci, aqueductal CSF flow void sign, medial temporal lobe atrophy, WM changes, mean temporal horn width (mTHW), EI-x, CA, and the modified cella media index (mCMI) (33). They found that NPH diagnosis was more likely in patients with higher disproportion of the SF-level SS as compared to the supra-SF SS space, and narrower THs (lower mTHW) but concluded that none of the radiological markers could predict shunt response. Given that this study only included patients with EI-x > 0.3, the finding of narrower THs in NPH may be due to patients with atrophic enlargement of the TH being grouped in the non-NPH group.

A quantification of DESH on CT scans was proposed using the SILVER index defined as the ratio of the SF area to the SS area at the vertex, which had an AUC of 0.9 in distinguishing NPH from controls, but there was no significant difference between shunt responders and non-responders (35). Another retrospective study that tried to relate various manually evaluated MRI measures including notable ones such as DESH (HC sulcal tightness and SF dilation), the CA, EI-x, maximum 3 V width, maximum width of the TH, maximum anteroposterior diameter of the 4 V, aqueductal flow-void sign, and inter-hemispheric fissure width found no significant differences between shunt responders and non-responders (56). Consolidating several markers of ventriculomegaly and CSF space morphology, Kockum et al. (37) proposed the iNPH Radscale in 2018. They demonstrated that this measure showed consistency when measured on MRI and CT (57), and had a positive correlation with expert radiologist evaluations and classified shunt-responsive NPH patients from healthy controls (50) using CT scans. However, there is also evidence that the Radscale cannot predict shunt response amidst symptomatic individuals and recommended against its sole for shunt-surgery selection (58).

A novel measure called the Splenial Angle (SA), defined on Diffusion Tensor Imaging (DTI) MRI Fractional Anisotropy maps as the angle subtended by the forceps major of the corpus callosum (CC) on axial slices, to capture the effect of ventricular distention on the CC at the ML in probable NPH was shown to have high sensitivity in distinguishing it from controls (30). Left ear extinction in NPH possibly associated with the upward elevation and thinning of the CC was found to be alleviated among patients with NPH post shunting, indicating the range of impairments that may be reversed with surgery (59). Confirming the previous findings of loss of WM integrity in NPH on CT scans, a recent study showed that the ratio of GM density at the thalamus to the WM density at anterior periventricular regions was lower in NPH and could be used to distinguish it from healthy controls. Even though their NPH cohort included definite NPH patients, they were unable to demonstrate the utility of this measure in distinguishing between shunt response in symptomatic patients (51). Hippocampal atrophy was shown to correlate with patients who have higher rates of cognitive impairment in NPH (53). But GM degradation had not been detailed in NPH until recently. Lv et al. (52) compared definite NPH patients with controls using MRI scans and found regional variations in GM volume that were significantly different in the NPH group. GM volume was lowered in specific temporal areas, thalamus, hippocampus, and the cerebellum. Contrarily, it was increased in medial and parietal regions. Global reduction in WM volumes and increase in CSF volumes were also found (52).

A 2013 study on MRI scans of 16 NPH patients who improved after shunt surgery showed that recovery after surgery was correlated with lower brain deformation before surgery as seen on MRI (60). They captured the ratio of ventricular and SF enlargement to the HC/ML tightness in a single measure of deformity, reflective of DESH, which was shown to improve post-surgery. It is important to note from this study that there may be a threshold to structural damage which may indicate irreparable loss when breached. And as new therapies emerge, it would be critical to accurately identify patients with this type of reversible dementia to optimize care for them. This is where differential diagnosis for NPH including the accurate identification of AD and PD comes to the forefront. The prominent features capturing the difference between NPH and AD/PD are summarized in Table 4.

**Table 4:** Structural imaging markers for differentiation of NPH from AD and PD.

Disease and comparison groups, Reference	Structural marker	Imaging modality
229 probable NPH, 161 non-NPH; All had EI-x > 0.3 (33)	mean width of the TH: NPH < non-NPH; Ratio of CSF volume in SS at the level of and superior to the SF: NPH > non-NPH	CT and MRI
19 probable NPH, 19 NC, 19 AD, 19 PD (30)	SA: NPH < AD, PD, NC	DTI MRI
17 definite NPH, 17 AD (61)	LV volume, 3 V volume: NPH > AD; Perihippocampal fissure volume: NPH < AD	T1 and T2 MRI*
34 probable NPH, 34 AD, 34 NC (62)	GM density in the parietal (lt, mdl), frontal regions (lt, mdl) except in the pre- and post-central gyri, and precuneus, volume of ventricles, sylvian fissure, and basal cisterns: NPH > AD, NC; GM density in thalamus, caudate, and perisylvian fissures: NPH < NC; GM density in thalamus: NPH < AD	MRI – T1 and T2*
13 probable NPH, 15 AD, 15 NC (63)	FA of the hippocampus: AD < NPH < NC; MD of the hippocampus: AD > NPH > NC	DTI MRI*
18 probable NPH, 11 AD, 11 PDD, 19 NC (64)	FA of the CST: NC, PDD, AD < NPH; Axial Diffusivity of the CST: NC, PDD, AD < NPH; Leukoaraiosis in the PV space: NPH > NC, PDD	DTI – MRI*
20 definite NPH, 20 AD, 20 PD (65)	FA of the supratentorial WM: NPH < AD, PD; MD of the supratentorial WM: NPH > AD, PD; Ratio of 3 V, LV volumes and supratentorial ICS: NPH > AD, PD	DTI – MRI*
22 probable NPH (12 definite NPH), 20 AD, 20 NC (66)	Volume, Cross-Sectional Area of the fornix: NPH < AD, NC; Length of the fornix: NPH > AD, NC; FA of the fornix: NPH < NC	DTI – MRI*
28 probable NPH, 28 AD, 20 NC (67)	FA of the anterior coronal radiata (bl), CC, Superior longitudinal fasciculus (bl), Posterior thalamic radiation (bl), External capsule (bl), Middle cerebellar peduncle: NPH < AD, NC; MD at the same locations: NPH > AD, NC	DTI – MRI*
17 probable NPH, 14 AD, 17 NC (68)	FA, MD, RD of cortico-fugal fibers from the frontal and parietal cortex: NPH > NC; FA of the CC (splenium): NPH < NC; RD of the CC (splenium): NPH > NC; FA, MD, RD of the corona radiata in the PV fibers from the frontal and parietal cortex: NPH > AD	DTI – MRI*
34 probable NPH, 34 AD, 34 NC (69)	CA: NPH < AD, NC	T1 – MRI
36 definite NPH, 34 AD, 36 NC (70)	CA: NPH < AD, NC; EI-x: NPH > AD, NC; GM volume: NPH > AD; LV + 3 V volume: NPH > AD, NC; Hippocampus volume: NPH > AD, NC	T1 – MRI*
42 probable NPH, 61 AD, 65 NC (27)	ACA: NPH < AD, NC	T1 – MRI
10 probable/definite NPH, 18 PD, 10 NC (71)	FA of anterior thalamic radiation, forceps minor and major, superior longitudinal fasciculus, CST: NPH < PD, NC; EI-x: NPH > PD, NC	DTI MRI*
5 definite NPH, 5 P, 10 AD, 10 NC (72)	Ventricular Volume, Ratio of Ventricular Volume and ICS volume: NPH > AD, PD, NC; Ventricular Volume/Cortical thickness: NPH > AD, PD, NC	T1 – MRI*
15 definite NPH, 17 AD, 18 NC (73)	Ventricular volume and GM volume: NPH > AD; Ventricular volume: NPH > NC	T1 – MRI*
15 probable NPH, 15 AD, 15 NC (74)	*VS*, SF volume: NPH > AD, NC; HC, ML volume: NPH < AD, NC	T1-MRI*
30 NPH, 10 NPH + AD, 18 AD, 26 NC (38)	Volume of the basal cisterns and SF: NPH, NPH + AD, AD > NC; Volumes of CSF in frontal and parietal convexity SS and upper SS: NPH, NPH + AD < AD; EI-z: NPH, NPH + AD > AD; CA, BVR: NPH, NPH + AD < AD	T2 – weighted MRI*
24 NPH, 22 AD, 40 NC (26)	simpCA: NPH < AD, NC	T1 and T2/FLAIR MR sequences
19 definite NPH, 24 AD, 18 PD, and 14 NC (75)	*VS*, SF volume: NPH > AD, PD, NC; HC, ML volume: NPH < AD, PD, NC; Ratio of *VS*, SF volume and HC, ML volumes: NPH > AD, PD, NC	T1 – MRI*
12 possible NPH (10 probable), 14 AD, and 17 NC (76)	*VS*, SF volume: NPH > AD, NC; HC, ML volume: NPH < AD, NC; Ratio of *VS*, SF volume and HC, ML volumes: NPH > AD, NC	T1 – MRI*
42 probable NPH, 32 AD, 24 NC (77)	CA: NPH < AD, NC; GM volume: NPH > AD; CSF volume: NPH > AD, NC; WM volume: NPH < AD, NC; Ratio of WM to TIV: NPH < AD, NC; Ratio of CSF to TIV: NPH > AD, NC; Ratio of GM to TIV: NPH < NC	T1 – MRI*

“*” indicates that (semi) automatic assessment methods were used. NC, Normal Controls.

## 4 Alzheimer’s dementia

Alzheimer’s Dementia (AD) is best characterized by the abnormal presence of extracellular amyloid plaques and intracellular neurofibrillary tangles (NFT) of protein tau which leads to a loss of synapses and cell death (78). In the US, AD is prevalent among 6.5 million people (79). Studies have shown that amyloid beta plaques start accumulating in abnormal clumps called oligomers and fibrils in the early stages which is correlated to downstream accumulation of NFT (80). Activated microglia that are unable to keep up with debris clearance cause chronic inflammation and cell death leads to atrophy. In early stages, the medial temporal lobe involving hippocampus, entorhinal cortex, amygdala, and para-hippocampal cortex seem to be most affected along with global atrophy involving the frontal, temporal, and parietal lobes excluding the sensory-motor cortex and occipital regions. Basal ganglia regions like the caudate, putamen, pallidum, and nucleus accumbens also show atrophy (15). Clinical symptoms mainly include loss of memory, decreased executive function, language deficit, loss of vision, and gait instability (78). Diagnosis is based on physical exam, evaluation of patient history, memory, cognitive, and neurological functions along with radiographic imaging. Therapeutic solutions for AD like cholinesterase inhibitors are commonly used to alleviate symptoms. While there is no complete cure for the disease yet, a drug which was shown to remove amyloid plaque deposits in the brain was approved by the FDA and several others are being investigated (79).

### 4.1 Structural imaging markers for the differentiation between NPH and AD

The medical research community has directed significant efforts to solving the problem of accurately distinguishing between NPH and AD using neuroimaging markers, in the hope that patients with reversible dementia do not get sentenced to a hopeless diagnosis of AD and miss their chance at a better quality of life. Identifying the structural effects of NPH and AD accurately may also find application in predicting patients with poorer shunt outcomes in NPH patients with comorbid AD (81). Expectedly, MRI-based markers and methods have dominated and emerged successfully in this quest. The CSF flow void sign on T2-weighted MR sequences which appears in the cerebral aqueduct was one of the earlier signs of impaired CSF dynamics in NPH, and it was shown to classify it from AD (82). This was also substantiated quantitatively by demonstrating a higher aqueductal CSF flow rate in NPH as opposed to AD, using phase contrast MRI, a few years later (83). However, even though CSF hydrodynamics is impaired in NPH, there is also evidence that it may not provide distinction from AD (84).

Signs of structural integrity loss in specific anatomies affected by the NPH pathology have been promising in segregating AD pathology. Holodny et al. (61) identified the dilation of the peri-hippocampal fissure as a distinctive anatomical marker between NPH and AD, along with the LV and 3 V sizes, as seen on MRI. Potentially indirect effects of ventriculomegaly like the narrowing of the posterior cingulate sulcus as compared to the anterior part and a concave upper midbrain profile, apparent on MRI, was shown to be more likely in NPH as opposed to AD but it was not tested for its sensitivity in classifying NPH from AD (54). While these studies set a promising avenue, extracting features from these specific neuroanatomies on MRI and CT requires significant manual intervention and/or preprocessing in the form of region-of-interest definitions. GM density on T1 and T2 MRI was shown to be significantly higher in the precuneus, frontal and parietal regions (medially and laterally, except around the central sulcus potentially spared due to dilation from aging) when NPH was compared to AD and normal controls. When NPH was compared to normal controls, it was significantly lower in the thalamus, caudate, and perisylvian fissure, but only so in the thalamus when compared to AD. It also revealed the enlargement of the ventricles, SF, and basal cisterns in NPH versus AD and normal controls (62).

WM abnormality has gained importance as a structural marker in differentiating NPH from AD. A 2010 study argued for the use of DTI to detect microstructural integrity of WM in the hippocampus using fractional anisotropy (FA) and mean diffusivity (MD) as more sensitive measures in detecting NPH from AD, as opposed to its whole volume (63). FA and axial diffusivity values measured on DTI MRI at the CST, distention of which is thought to cause gait impairment in NPH, were shown to be highly sensitive in detecting probable NPH from AD, PD with Dementia, and healthy controls (64). In the same year, another study found that higher MD coupled with lower FA in the supratentorial WM of the brain was indicative of NPH versus AD and PD (65). The SA which captures the effect of distention of LVs on the forceps major of the CC (30) was also shown to differentiate between NPH and AD.

In a very insightful finding on DTI, it was demonstrated that the WM structure fornix had reduced volume and cross-sectional area in NPH patients but longer compared to AD and normal controls (66). The FA value in this structure was also lower in NPH as compared to normal controls. Revealing structural damage to the fornix due to LV enlargement, this finding illustrates the power of structural imaging to capture the degenerative effects of NPH on the brain. Kang et al. (67) provided a more anatomically localized insight into WM structural integrity by showing lower FA and higher MD bilaterally in the anterior corona radiata, posterior thalamic radiation, superior longitudinal fasciculus, and external capsule of NPH patients as opposed to AD patients, as well as in the CC and the middle cerebral peduncle. They showed the FA was lowered in the splenium of the CC and right external capsule in NPH patients with higher gait disturbance. Another study of WM integrity using DTI parameters like MD, FA, radial diffusivity (RD), and axial diffusivity showed a higher FA, RD, and MD in the corona radiata in periventricular fibers from the frontal and parietal cortices in NPH as compared to AD (68).

When attention is shifted to the structural degenerative markers that are seemingly similar in NPH and AD, ventriculomegaly emerges in the top spot. The CA has long been established as a direct marker that can capture if the ventricular enlargement is due to true hydrocephalus in NPH or a compensated enlargement due to atrophy in AD on MRI (69). This finding has been extensively validated, and even by more recent studies. The simpCA was also shown to be significantly lower in NPH as opposed to AD (26). Using manually annotated CA and EI-x measures on MRI images, an AUC of 0.96 was reported in distinguishing 36 definite NPH patients from 34 AD patients and 36 healthy controls (70). Even though a cut-off of 0.3 is usually recommended for the EI-x, it was found that a cut-off of 0.32 for the EI-x and 100^0^ for the CA maximized diagnostic accuracy, and the performance metrics were reported based on this classification. The EI-x cut-off of 0.3 has also been contested with a better proposal of age and sex specific values pointing to higher sensitivity (85). The anterior CA (ACA) was proposed and tested against the conventional CA, and EI-x on MRI scans in distinguishing NPH from AD and healthy controls (27). While it was significantly lower in NPH as compared to AD and healthy controls, it did not outperform the CA in diagnostic accuracy. A subsequent study of its association with gait impairment showed correlation with pre-surgery symptoms and post-operative improvement (86). Overall, we found that numerous distinctive features of NPH versus AD have been studied, but MRI is predominantly used as opposed to CT.

## 5 Parkinson’s disease

Parkinson’s disease (PD) is primarily diagnosed in a clinical setting through thorough examination of neurological symptoms that have motor manifestations like tremor, impairment of gait, arm-swing, balance, postural stability, and facial expression, and non-motor manifestations like impairment of behavior and cognition. Definitive diagnostic tests using blood, CSF, or imaging biomarkers do not currently exist (87). The manifestation of dementia in PD (PDD) patients (16) makes some patients more susceptible to misdiagnosis. There are reports that indicate that more than 15% of patients with PD may be misdiagnosed (88), with prominent misdiagnoses including AD (16). NPH may also be misdiagnosed as PD due to parkinsonism (89). Early diagnosis of this disease is challenged by the late manifestation of its defining motor symptoms, heterogeneity in clinical presentations, underlying mechanisms of its subtypes, age at onset, rate of progression, and response to treatment. This is also reflected in the heterogeneity of imaging features which correlate with symptomatic presentation (87).

As a direct consequence of the fact that the source of this synucleinopathy is known to primarily affect the substantia nigra (SN) and basal ganglia, and its microscopic magnitude of origin, imaging markers from structural imaging which rely on the scatter of external radiation like CT and MRI are not recommended for assessment of PD. Rather, functional imaging markers that directly correlate with the function at cellular levels by reflecting the uptake of specific radioactive tracers are the most viable option for detection, especially in early stages. In support of this claim is an article from Dalaker et al. (90) who found that global markers like atrophy and white matter hyperintensities were not significantly distinct in early PD as compared to healthy controls. Recently, Bae et al. (91) reviewed the degenerative markers of the SN on advanced imaging techniques like Nigrosome and Neuromelanin Imaging (NMI) using high field MRI techniques, Quantitative Iron Mapping (QMI), and Single Photon Emission Computed Tomography (SPECT). They highlight studies which have shown highly sensitive classification of PD when iron deposition in the SN driven by dopaminergic cell loss is used as a biomarker. In support of high field and neuromelanin sensitive MRI is a review from 2019 by Prange et al. (92) and from 2016 by Pagano et al. (93) Near normal or diffuse cortical atrophy is associated with PD (94), which raises the possibility of no visible markers of degeneration on CT or MRI. We investigate this further by providing the following review of structural imaging markers in PD and those that can distinguish it from NPH on MRI and CT sequences.

### 5.1 Structural imaging markers for the differentiation between NPH and PD

The presence of parkinsonian symptoms is not an uncommon occurrence in NPH (95), with case reports that were made as early as 1983 (96) which also recognized that it did not negatively impact shunt outcome. A 1994 report presented insight into the pathophysiological mechanisms of co-occurring hydrocephalus and parkinsonism manifesting as impairment of the nigrostriatal or neural circuits traversing the cortex, striatum, pallidus, and thalamus (97). Their proximity to the enlarged ventricles may introduce mass effects and ischemic changes. According to Akiguchi et al. (89) WM lesions and parkinsonism symptoms (prevalent in 71% of patients) in NPH were reversed after shunt surgery. Given this, differentiating NPH from PD, with which it is so frequently mistaken, is of paramount importance.

DTI has been shown to distinguish between NPH and PD using FA measures at the forceps major and minor, anterior thalamic radiations, CST, and superior longitudinal fasciculus by Marumoto et al. (71). Interestingly, they also found that the EI-x was significantly higher in NPH as compared to PD (100% sensitivity). The anterior thalamic radiation FA measure demonstrated higher specificity as compared to the EI-x in distinguishing NPH from PD. Ventricular dilation and atrophy have been demonstrated in PD (greater at baseline in those with dementia) on MRI (98), so Marumoto et al.’s (71) finding of a higher EI-x in NPH versus PD may suggest that ventricular enlargement in PD may not be as high as NPH. Measures of WM structural deformation and integrity measured through DTI have also been shown to distinguish between NPH and PD. The SA introduced by Chan et al. (30) was significantly higher in NPH versus PD. Kanno et al. (65) found lower FA and higher MD in the supratentorial WM in NPH when compared to PD. Visual evaluation of periventricular WM demonstrated significantly higher leukoaraiosis in NPH when compared to PDD and healthy controls (64). There are a limited number of studies which have examined the structural differentiation between NPH and PD, which may be a general reflection of the fact that structural imaging markers are discouraged in the assessment of PD.

Burton et al. (16) demonstrated GM and overall volume loss in PD as compared to healthy controls localized to the bilateral temporal and occipital lobes, thalami, right putamen, caudate tail, and middle-inferior frontal gyri. A review from 2017 highlights the structural markers of PD on MRI, with notable volumetric changes seen in the basal ganglia, GM volume loss in the frontal lobe, cingulate, temporal gyri, hippocampus, and loss of cortical thickness in specific frontal and occipital areas as compared to controls (99). Adding to the nuance which can be picked up on structural imaging, a recent study using MRI scans showed a difference in the amount and pattern of GM and cortical atrophy between patients with PD-without-dementia and healthy controls (100). As expected, pronounced structural effects were found in more severe manifestations of the disease. With a comprehensive classification system that factored clinical, demographic, and symptomatic presentations to categorize patients into mild and moderate–severe groups, they found significant differences only between the moderate–severe group and the healthy controls. The CC volume in the mid-anterior and central regions were found to be reduced in PD patients on MRI as compared to healthy controls by Goldman et al. (101). Shape changes and volume loss in the putamen, and shape changes in the caudate were shown to distinguish between PD and healthy controls (102). In a first attempt of its kind, a study from 1985 found that the size of the CSF spaces was increased in patients with PD when compared to normal controls on CT scans (103). Asymmetric ventricular enlargement was reported in PD using MRI scans (104), and later verified on 17 PD patients (105). Longitudinal atrophy and ventricular enlargement measured on MRI has been reported even in PD patients with dementia (106). Amidst the discouragement of structural imaging in the assessment of PD, textural features derived from first and second order grayscale/intensity statistics on T1-MRI scans were shown to be significantly associated with clinical features of PD by a 2021 study (107). In this highly welcome development, they showed that changes in the nigrostriatal pathway in early stages of PD could be captured through structural images, which was supported by correlation with motor symptoms. With these insights into the structural degradation in PD as opposed to normal controls, there is a considerable knowledge pool of structural markers that may be tested for their differentiative capacities from NPH.

## 6 Computational methods for the detection of NPH and its differentiation from AD and PD

Early adopters of semi-automatic algorithms included an effort to extract volumetric ventricular sizes and cortical thickness for distinction of NPH from AD, PD, and normal controls using MRI, and advocated for the ratio of ventricular size and cortical thickness as a better feature to distinguish between definite NPH (*n* = 5), from the rest, especially as ventricular volumes in NPH and AD may overlap (72). This problem has also been countered by considering the distribution of (normalized) CSF volumes rather than using it as a global measure, as shown by many studies so far. Voxel-based morphometry (VBM) has long been established as a reliable computational tool in MRI studies to assess structural markers of NPH. Yamashita et al. (76) analyzed CSF distribution using VBM on MRI scans and showed significant distinction between NPH (*n* = 12 with 83% probable NPH and 17% definite NPH) when compared to AD and normal controls in terms of LV/SF volumes, and HC/ML volumes. In a measure that reflects DESH, their analysis also showed that the volume of CSF in the LV and SF areas as compared to that in the HC and ML areas was higher in NPH as compared to AD and normal controls. Subsequently, they showed similar trends in those features on a definite NPH cohort, and when it was compared to PD patients (75).

Ishii et al. (74) developed an automatic volumetric CSF segmentation method on T1-MRI, which provided localization into the SF, HC, and ML sulci, as well as the ventricular spaces. They demonstrated the validation of previously known findings that enlarged ventricles and SF, and tight HC sulci were evident in a group of 15 probable NPH patients as opposed to AD and healthy controls. In a more targeted and automatic effort to segment GM, WM, and CSF spaces on T1-MRI, ventricular and GM volume, and gender were found to distinguish shunt-responsive NPH from AD and normal controls (73). Ellingsen et al. (108) developed a segmentation and labeling software to compartmentalize the ventricular system (into lateral, third, and fourth), referred to as RUDOLPH on MRI at a time when limited or no efforts were made for automatic segmentation of highly deformed ventricles. Yamada et al.’s studies on indices characterizing CSF spaces for the optimal distinction of probable NPH from healthy controls (29), AD, and NPH-and-AD cohorts on T2-MRI (38) were also based on semi-quantitative approaches relying on automatic segmentation of brain tissue. In a recent study of CSF distribution, the normalized WM and CSF volumes derived using VBM, along with the CA were shown to provide improved distinction between probable NPH and AD using MRI sequences when compared to the CA alone (77).

Peterson et al. (55) used an automatic segmentation method on T1-MRI to quantify volume in deep subcortical GM structures and showed reduced volume of the caudate, putamen, thalamus, nucleus accumbens, hippocampus, and pallidum in 14 NPH patients as compared to 8 healthy controls. Efforts apart from volumetry have also been made to extract nuanced MRI-based features. Statistical parametric methods using MD histogram analysis from DTI images have also been shown to distinguish between probable NPH and AD/PD/normal controls with minimal manual intervention (109).

Linear and interpretable measures of ventriculomegaly have also been computationally assessed on MRI. Borzage et al. (7) developed an automated methodology to extract CA from MRI from the Open Access Series of Imaging Studies (OASIS) and Alzheimer’s Disease Neuroimaging Initiative (ADNI) databases. With a high ICC of 0.9 between the automated measure and expert annotations on 281 images and an innovative approach to coronal pitch correction for image standardization, they showed the utility of data-driven applications in objective evaluation of diagnostic standard features. CT based methods for automatic assessment of interpretable measures have been limited. An automated image-processing methodology to extract the EI-x from CT images reported a correlation coefficient of 0.983 between the automated measure and expert annotations among 44 subjects (12 NPH). However, they applied nonlinear registration for image standardization which would render the brain width as a constant and affect the measurement of the EI-x (110).

Deep learning-based methods have shown promise in the objective and automatic assessment of NPH using structural imaging modalities. Again, MRI dominates this field of investigation. To enable the automatic segmentation and parcellation of the ventricular system in patients with severe ventriculomegaly due to hydrocephalus, Shao et al. (111) developed a deep learning model named VParNet trained on MRI scans of NPH patients and healthy controls, which showed high agreement with expert annotations and outperformed state-of-the-art. However, they did not test the classification capacity of their model. Irie et al. (112) developed a 3D convolutional-ladder network, and showed that their model can distinguish between probable NPH (*n* = 23), AD, and controls with an accuracy, sensitivity, and specificity of about 90% using MRI scans. By showing regions like the periventricular spaces, hippocampus, and THof the LVs being highlighted in the activation maps sensed by the network on some successfully classified scans, they demonstrated agreement with previous neuroanatomical findings in their findings.

Deep learning methods to assess NPH on CT scans have also emerged recently. Considering an EI-x ≥ 0.32 to be an indicator of hydrocephalus, a transfer learning scheme was applied on a large dataset of CT scans to show a classification performance with AUC of 0.93, sensitivity of 93.6%, and specificity of 94.4% in distinguishing hydrocephalus from normal controls (113). Automated segmentation of CSF, subarachnoid, and cerebral spaces on non-contrast CT scans of 27 patients with possible NPH, integrated with indirectly inferred connectome data, was shown to be as effective as the EI-x for prediction with a specificity of 85% and sensitivity of 86% (114). Haber et al. (115) recently developed a convolutional neural network (CNN) which was able to classify patients with definite improvement post-surgery as identified by the 2nd edition of the Japanese guidelines from healthy controls using CT scans setting a promising precedent in the application of deep learning to CT scans in NPH assessment. Further studies are required to study the potential of deep learning in assessing imaging markers that can predict positive and objective shunt response in symptomatic NPH and distinguish it from its mimics.

## 7 The chronic effects of traumatic brain injury

TBI casts a pervasive shadow, affecting an estimated 27 to 69 million individuals globally each year, with each case carrying significant chronic consequences (116, 117) and pathophysiological connections to various neurodegenerative diseases including NPH, AD, and PD. Its impact can range from concussions and diffuse axonal injury (DAI) which are harder to detect on structural imaging to extra-axial and intracerebral lesions that appear prominently. While acute injury is easily identified on CT and treated surgically, detecting (and differentiating between) its chronic degenerative consequences including atrophy and hydrocephalus which is crucial for optimal management may be challenging.

Subdural hemorrhage (SDH) refers to bleeding that occurs in the subdural space of the brain. And chronic SDHs (cSDH) represent these lesions which may evolve bilaterally in patients with symmetric cranial vaults, but most frequently occur on the side with higher frontal or occipital convexity. They may also be seen at interhemispheric locations in some cases (118). Even though intracranial hypotension and defective coagulation can cause cSDH, mild trauma to the head remains the predominant cause (119). Rupturing bridging veins in the subdural space following trauma may cause hematomas with varying cellular and vascular compositions as they evolve (118). It mostly affects older patients and is diagnosed with CT even though MRI might be more sensitive in isodense cases. Cerebral atrophy has been shown to be a risk factor for cSDH using volumetric analysis of CT scans (120), as well as being a prominent chronic consequence of it (121). Progressive volume loss in cSDH, at rates higher than dementia, have also been reported using volumetric analysis of CT scans (122). While hydrocephalus is also reported to be a risk factor for the development of cSDH following a minor head injury (123), there has been no compelling investigation into its development post cSDH.

Subarachnoid hemorrhage (SAH), which refers to bleeding in the space between the arachnoid and pia mater of the brain, is associated with secondary hydrocephalus (124) and atrophy (125). NPH was shown to develop in 21% of patients within a month following SAH, and improvement was noted in 85% of patients who underwent shunt surgery (126). While SAHs can occur due to nontraumatic causes, it is also estimated to occur in 33–60% of patients with moderate to severe TBI (127). Dilation of the LVs, as assessed with linear measures such as the bicaudate index and dilation rate assessed as change in volume over time on CT (124), atrophy measured indirectly as the modified cella media index (mCMI) and normalized CSF volume on T1-MRI (32), and parenchymal volume loss assessed through volumetric methods (128) are known structural markers of nontraumatic/spontaneous SAH. Hydrocephalus also occurs in traumatic SAH (13, 129, 130) which are usually found in the sulci at the convexities (131). Tian et al. (129) found that it may be correlated with intraventricular bleeding, severity of injury, thickness, and location of the lesion. Overall, we found that there are limited efforts to characterize and differentiate between the structural markers of hydrocephalus and atrophy following traumatic SAH. The prominent features of atrophic and hydrocephalic degradation in cTBI are summarized in Table 5.

**Table 5:** Structural imaging markers of cTBI.

Disease and comparison groups, Reference	structural marker	Imaging modality
PTH – 29 shunted, 807 not shunted (132)	Higher EI-x, mFHI at discharge correlated with PTH needing shunt	CT
7 without DNL, 8 with DNL (39)	DNL: Acute SDH with contusion Sudden increase in BCVI over 18% after 1 month of injury, Higher hemispheric CVI ipsilateral to injury, Focal hypodensities and prominent sulci ipsilateral to injury WM damage	CT
14 mild or moderate TBI (133)	Rate of decline in volume of brain parenchyma: cTBI > NC	MRI*
21 with mild TBI (134)	High prevalence of brain lesions, correlated with volume loss in whole brain parenchyma after 6 months CSF volume	T2 – MRI, FLAIR, SPECT*
54 with mild TBI, 31 NC (135)	Mean cortical curvature in 25% of (bl) 31 sulcal and 29 gyral regions: cTBI > NC; Deep GM structures most affected	T1 – MRI*
86 with cTBI, 46 NC (136)	Fornix and Hippocampal atrophy: cTBI > NC	MRI*
118 cTBI, 136 NC (137)	CSF volume in the TH and subarachnoid sulci, TH dilation, hippocampal atrophy secondary to WM damage: cTBI > NC	T1 and T2 – MRI*
27 cTBI, 12 NC (138)	Cross Sectional Area – Posterior Cingulate Gyrus, Corpus Callosum, Thalamus: cTBI < NC; Cross Sectional Area – Lateral Ventricle: cTBI > NC; Whole brain volume: cTBI < NC; Lateral ventricle volume, ratio of lateral ventricles to whole brain volume: cTBI > NC	T1, T2-MRI*
24 boxers (repetitive mild trauma), 14 NC (139)	Whole brain volume loss, Degradation of the septum pellucidum, Periventricular and subcortical WM disease, diffusion constant of the brain: cTBI > NC	T1, T2, DTI – MRI*
25 cTBI, 22 NC (140)	Whole brain, WM, Cortical (precuneus, parietal cortex, and paracentral lobule) and subcortical (notably in the CC, amygdala, hippocampus, putamen, and thalamus) volumes: cTBI < NC; CSF and LV volumes: cTBI > NC	T1 – MRI*
28 cTBI, 22 NC (141)	Whole brain, WM structures of the cingulate, GM of the precuneus volumes: cTBI < NC	T1 and T2 – MRI*
50 cTBI, 50 NC (142)	FA in the frontal cortex: cTBI > NC	T1, T2, DTI – MRI*

“*” indicates that (semi) automatic assessment methods were used. NC, Normal Controls; PTH, Post-Traumatic Hydrocephalus; DNL, Delayed Neuronal Loss.

### 7.1 Structural imaging markers of hydrocephalus and atrophy in cTBI

Hydrocephalus is known to be associated with TBI, irrespective of the presence of traumatic lesion (143). Therefore, it is pivotal to differentiate ventriculomegaly resulting from hydrocephalus and atrophy, as the former may be surgically treated. A call for this was made by Marmarou et al. (13) as early as 1996. In their study, 44% of patients with a severe head injury displayed ventriculomegaly (EI-x > 0.3), with 20% of them indicative of hydrocephalus as per CSF dynamics (monitored as intracranial pressure and CSF outflow resistance). Patients with an SAH showed a higher incidence of ventriculomegaly (80%) than those without (30.8%), and hydrocephalus was detected through CSF dynamics in both groups (50 and 9.1%, respectively). They described a methodology which combines the EI-x and CSF dynamics to classify patients as having high pressure hydrocephalus, NPH, or ventriculomegaly secondary to atrophy, and suggested shunting in the high pressure and normal pressure groups. The need for this distinction was reemphasized by Guyot and Michael (14) who also recognized that shunt-response was dependent on the distinction between symptomatic-hydrocephalic and atrophic ventriculomegaly (14), with the former group faring better.

A later study proposed a noninvasive selection criterion for shunt surgery in this group. 45% of patients with a severe TBI were found to have post-traumatic hydrocephalus, and it correlated with decreased perfusion in the temporal lobes, as seen on SPECT imaging 2–4 months after injury was found to improve post shunting (144). This imaging marker was dependent on functional imaging, but a valuable insight from this study is that even though hydrocephalus may develop within 3 months after injury, ventriculomegaly secondary to atrophy may not be seen until 6 months. This emphasizes the importance of longitudinal imaging to assess the nature of ventriculomegaly in chronic stages. In a recent finding and larger study (n = 836), the incidence of post-traumatic ventriculomegaly was found to be 46% in TBI patients (132). 3.5% of the patients in the study received shunts (post-traumatic hydrocephalus), with an improvement that was seen in 66%. The patients selected for shunting all displayed ventriculomegaly, but clinical decision based on symptoms was a predominant factor in their selection. Those with low pressure hydrocephalus had better outcomes, and the EI-x and modified frontal horn index (mFHI) were found to be higher in the acute phase for those who developed post-traumatic hydrocephalus.

In a study following patients with severe TBI, 88% displayed ventriculomegaly, and in 1–4 months after injury, a distinct atrophy/degradation termed as delayed neuronal loss (DNL) was observed in about 50% of patients through the bicaudate cerebroventricular index (BCVI) on CT scans. This measure progressed rapidly after 1 month of injury, indicating the sudden manifestation of DNL. Interestingly, acute SDH with contusion was the only contrasting finding between patients with DNL and those without it (39). Those patients’ CT findings also included the enlargement of the ipsilateral ventricle as measured by the hemispheric cerebroventricular index (CVI), a sudden appearance of a hypodense focal area ipsilateral to injury (confined to areas of the middle and anterior cerebral arteries) and WM damage as opposed to preserved cortical structures.

Whole brain atrophy has been an established marker of degradation in chronic TBI. Using analysis of MRI, it was found to occur post 11 months of injury and was correlated to loss of consciousness (133). In a prospective study of young patients with mild TBI, even though their neurocognitive profiles were normal, it was found that lesions on MRI (T2-weighted) were associated with whole brain atrophy 6 months post injury, emphasizing the essentiality of radiological follow-up in the post-traumatic course after an initial injury is detected (134). Another structural marker which was found to be distinct in mTBI was the mean cortical curvature of various bilateral cortical structures as measured on MRI. This feature is different from the global volumetric ones, in the aspect of quantifying region-specific pattern of atrophy (135).

Evidence of degradation in specific cortical and subcortical structures has also been abundantly described over the years. Another study which conducted MRI analysis of TBI subjects after 2 months of injury found atrophy in the hippocampus and fornix as opposed to controls (136). An MRI-based tissue specific and volumetric analysis of the temporal lobe structures including the SF, hippocampus, THs of the LVs, temporal gyrus and sulcus, and temporal WM stem, showed that CSF volume was increased (in the THs, subarachnoid sulci) which was more related to WM damage than GM in TBI as opposed to controls (137). They also found that the TH dilation was more related to WM damage than hippocampal atrophy.

At a time when it was thought that medial structures were not as affected from TBI as compared to the frontal and temporal regions (which are more susceptible to TBI insult), an MRI based study showed that the cross-sectional area of the posterior cingulate gyrus (PCG), CC, and thalamus was reduced, and that of the LVs was increased in TBI patients as opposed to controls. As WM from the frontal, temporal, and hippocampal areas connect with the PCG, the authors suggested that these localized structural markers may capture trans-neuronal damage in TBI. The total brain volume was decreased, and the LV volume, and the ratio of the latter to the former were increased (138). A diffusion weighted imaging study of boxers showed increased diffusion indicative of microstructural damage, and MRI analysis (T1/T2/DWI/FLAIR) indicated age-inappropriate volume loss, degradation of the septum pellucidum, periventricular and subcortical WM disease as compared to controls (139). About 8 months following a TBI with axonal injury, whole brain atrophy and increased CSF volumes, cortical (precuneus, parietal cortex, and paracentral lobule) and subcortical atrophy (notably in the amygdala, hippocampus, putamen, and thalamus) localized to specific regions were found by analyzing MRI (T1-weighted) scans using a semi-automated morphometric analysis (140). Post 12 months after mild TBI, global brain atrophy, loss in volume of specific WM structures of the cingulate and precuneal GM was detected using T1-MRI (141). Cortical abnormalities may also be detected using diffusion measures such as increased FA in mild TBI (142). While hydrocephalus and (GM, WM) atrophy are well studied structural consequences of cTBI, studies of structural markers that can characterize their interplay in producing an appearance of ventriculomegaly are lacking.

## 8 Computational methods to assess the chronic effects of TBI

Computational techniques like tensor-based morphometry (TBM) have been used on MRI (T1-weighted) scans to study localized volume loss in cTBI. WM structures including but not limited to the CC, subcortical structures like the caudate (middle and posterior) cingulate, thalamus, frontal and temporal neocortices, and the cerebellum, were shown to be affected after at-least 3 months of moderate–severe TBI as opposed to controls using this technology (145). Ventricular volume was also revealed to be enlarged. This pattern remained irrespective of the presence of macroscopic lesions. SIENA (146), an automated software for brain atrophy quantification on MRI was applied by Trivedi et al. (147), to show significantly higher brain volume decline in mild-to-severe TBI as opposed to normal controls. This software was also used to quantify longitudinal brain atrophy on MRI (T1-weighted) among severe TBI patients and revealed an association between higher rates of brain atrophy and injury severity, and that brain volume change was a better predictor of long-term functional outcome as compared to functional measures at 8 weeks post-injury (148). TBM revealed localized atrophy in the brain stem, thalamus, putamen, and WM structures like the cerebellar peduncles, internal and external capsules, CC, superior and inferior longitudinal fasciculus, and corona radiata as opposed to normal controls. A surface-based morphometry (SBM) study including DTI and high-resolution MRI revealed distinct cortical thinning, GM diffusivity, and loss of integrity in the pericortical WM in TBI patients (149). Developments in the use of computational tools for objective assessment of atrophy after mild TBI have also led to an FDA approved software. It was shown to reliably capture progressive volume loss, and demonstrated degradation in whole brain (parenchyma, CSF, WM), and regional (forebrain, cortical GM, cerebellum, brainstem) structures on T1-MRI as compared to normal controls (150). This tool was subsequently shown to be more sensitive to (progressive) atrophy and asymmetry detection than radiologist interpretations (151, 152). In a very interesting application of this tool to predict previous brain volumes based on current measures, the developers also showed that reliable estimates of brain volumes were obtained on normal subjects. And that TBI patients show rapid progression of cortical atrophy, as opposed to enlargement in the subcortical nuclei and infratentorial spaces, in the few months after injury. As in the case of distinguishing NPH from AD and PD, we found that computational methods to assess atrophy and hydrocephalus cTBI are predominantly MRI based, and deep learning based approaches are lacking.

## 9 Discussion

There are abundant examinations into features that can classify between NPH and asymptomatic controls, but those that predict shunt response in symptomatic NPH have not been established. Imaging studies have typically focused on predicting probable NPH due to the apparently high clinical value. Therefore, prediction of tap-test/lumbar drain responses using structural neuroimaging markers as a non-invasive alternative for a preliminary assessment of shunt response is a well-studied topic. CSF space morphological features, characterizing the narrowing of the HC/ML sulci and higher SS, enlargement of the SF and lower SS, and vertical expansion of the LVs have been shown to be highly discriminative in predicting tap-test response. The EI-z and DESH capture them in a straightforward and interpretable manner, but the latter would benefit from a computational formulation. Benedetto et al. (35) proposed the SILVER index as a potential solution. However, localized measurements of SS require manual annotation which inevitably introduces observer variability and higher computational cost. Features characterizing GM and WM integrity have also been shown to correlate with tap-test response, but nonuniformity in scanner calibration, noise, and poor soft tissue resolution may impact such density-based measures on CT scans, which is additionally limited by the need for manual annotation on both CT and MRI scans for localized anatomical measurements.

While discriminating the tap-test response is important, the low sensitivity of the tap-test itself toward shunt response (153) suggests that features optimized to predict tap-test response might reflect or exaggerate the low sensitivity. The performance reports of imaging markers as predictors of shunt response in NPH have also been mixed. Features like the iNPH Radscale have shown prognostic value in discriminating shunt responsive from healthy individuals, but there is no compelling evidence that it can predict shunt response in symptomatic individuals. Additionally, response to shunting may depend upon many factors like post-surgery care, presence of comorbidities, physical therapy, shunt complications etc. which need to be considered in future studies. Optimizing the sensitivity of radiographic evaluation to predict NPH related signal whether it is possible, probable, or definite might enable higher screening and lower cases of underdiagnosis.

Addressing the high levels of misdiagnosis in NPH is also crucial to identify and treat patients with this reversible dementia. While features for the distinction of NPH from AD have been well established, we observe that there is a lack of neuroimaging studies to distinguish NPH from PD using structural imaging. Perhaps due to heavy reliance on levodopa response in patients with suspected PD or clinical symptoms. Clinicians and researchers alike must recognize that this is a suboptimal approach which may put true NPH patients at unnecessary risk due to levodopa side effects and delay treatment with shunt-surgery. We have identified features that are discriminative of PD from controls, which may be tested for their application of distinguishing NPH from it. We encourage researchers to explore computational approaches to fully explore the potential of structural imaging in distinguishing between NPH and PD. Shunt surgery is also capable of relieving post-traumatic hydrocephalus. But research is needed to test the potential of structural imaging markers in selecting patients for surgery, as it is riddled with the problem of distinguishing between atrophy and hydrocephalus following TBI. Unfortunately, in more than 20 years post Marmarou et al.’s (13) advocacy for the use of CSF dynamics in patient selection for shunting in post-traumatic hydrocephalus, an alternative solution with noninvasive markers has not been found.

It is also important to distinguish Long Standing Overt Ventriculomegaly in Adults (LOVA), which is a chronic form of hydrocephalus, from NPH. Most cases are thought to arise due to aqueductal stenosis and may have symptomatic overlap with NPH. Patients with LOVA may also see clinical improvement with shunt surgery (154, 155). It is crucial to differentiate it from NPH as it may be extremely sensitive to pressure variations, which needs to be considered while evaluating surgical treatment (endoscopic third ventriculostomy for LOVA versus ventriculoperitoneal shunt for NPH) options. Characteristic neuroimaging finds of LOVA include (third and lateral) ventriculomegaly, decreased prominence of cortical sulci, macrocephaly, and expansion of the sella turcica due to compensatory mechanisms (156). In cases where LOVA occurs with an open aqueduct, where it may be differentiated from Late Onset Idiopathic Aqueductal Stenosis (LIAS), it becomes more crucial to distinguish it from NPH due to their clinical and symptomatic overlap (157). ICP monitoring in LOVA patients was shown to correlate with patient conditions pre- and post-surgery (158), and CSF dynamics was recommended to differentiate them from NPH patients (159). A noninvasive and accurate diagnostic score consisting of clinical features like age, presence/absence of the Hakim triad, headache, nausea/vomit, and neuroradiological features (evaluated on MRI) like the head circumference, EI-x, 3 V width, DESH, sellar bone distortion with the bulging of the 3 V floor was proposed recently by Palandri et al. (157) which was shown to classify probable NPH patients from LOVA and LIAS patients with a high AUC of 0.97, sensitivity of 95.1%, and specificity of 90.6%. Higher values of this diagnostic score correlates with higher EI-x, 3 V width, head circumference, 3 V floor bulging, and lower prominence of DESH which incorporates distinctive neuroradiological findings in LOVA as opposed to NPH. More research is needed to identify imaging markers which can differentiate shunt-responsive NPH from LOVA/LIAS.

Even though the most prominent structural impact of NPH except for WM abnormality can be visualized on CT, most research studies lean toward MRI. We identify some CT based markers which have shown good predictive performance, and further encourage researchers to not only adapt MRI-based features to CT studies, but also develop CT-specific features for this application. CT-imaging biomarkers, as affordable, timely, and accessible diagnostic solutions, with fewer contraindications than MRI may offer a hopeful prospect. Even though MRI is the preferred mode of structural imaging due to its higher soft tissue resolution as opposed to CT, the development of computational tools to extract volumetric, intensity, and texture-based CT-features may reveal the potential in characterizing structural degeneration in NPH and its differential diagnosis from AD and PD. CT is the preferred mode of evaluation for acute TBI, but the distinction between atrophy and hydrocephalus in cTBI is still heavily studied only on MRI. Advances in computational tools, particularly in machine (deep) learning and image processing, may hold the key to discovering novel CT-based markers, and objectively extracting diagnostic standard markers for these conditions.

A popular choice in semi-automatic image processing methods is to integrate domain knowledge through the volumetric characterization of brain regions that are known to be affected. While this has been successful on MRI, obtaining pixel-level ground-truth for segmentation on CT is not only expensive, but difficult to create by visual inspection. Moreover, brain regions are impacted differently in different neuropathologies and developing individual segmentation-based approaches to capture them, and their interactions can be challenging and quickly add complexity. Architectures from CNN models with the inherent capacity to learn feature representations at increasing levels of abstraction, residual networks that utilize skip connections to solve the problem of vanishing gradients in deep architectures, and the U-Net and its variants that optimally integrate local and contextual features have been limited in the assessment of NPH, and mostly on MRI. The potential of such models in solving other problems such as differentiation of (shunt-responsive) NPH from AD/PD, and atrophy/hydrocephalus in cTBI on CT scans should be tested.

Our article has a few limitations. It is a narrative review. Even though the search strategy was methodical, it was not exhaustive in terms of databases as we only included PubMed. We did not conduct a dedicated search for articles pertaining to computational methods which have been reviewed in this paper, but they were isolated from the search described in the “Search Methodology” section.

## 10 Conclusion

Better recognition of (normal pressure) hydrocephalus is paramount for the timely management of patients with potentially reversible dementia or brain injury. Despite a plethora of knowledge on the discriminative anatomical markers of NPH, only a few like the EI-x, CA, and DESH have notably been included in diagnostic guidelines. While prediction of tap-test/shunt response may offer most assistance to clinicians, discriminating hydrocephalic pathology from irreversible atrophy is necessary to identify patients who may be surgically treated. Anatomical markers that can accurately predict shunt surgical response in symptomatic NPH are yet to be reliably established, which may be complicated by amount of pathological progression prior to surgery, comorbidities, and post-surgical care. Longitudinal evaluation of anatomical markers correlated with symptomatic progression before and after surgery may shed light on expected recovery and planning treatment options. Additionally, there is a clear under-utilization of CT based markers in distinguishing NPH from AD, and an overall lack of studies assessing its structural differentiation from PD. Investigations into the distinction of the chronic effects of TBI, namely atrophy and (normal pressure) hydrocephalus, using structural imaging markers is also a crucially unaddressed area which may help to identify patients whose symptoms may be alleviated with shunt placement. Computational tools like image processing may help with objective measurement of features that are correlated with NPH pathology on CT; and advances in deep learning may also highlight explainable features with potential for accurate diagnosis. In emergency settings, smaller community care centers, and hospitals that may not have access to advanced imaging and the expertise to assess them, automatic methods for assessing CT scans for the accurate detection of NPH or post-traumatic hydrocephalus may be of immense value in recognizing patients with reversible symptoms. Through this effort, we urge researchers to untangle the web connecting these neurodegenerative conditions, offering hope to millions, and potentially preventing countless more from stumbling down the treacherous path of structural neurodegeneration.

## Author contributions

SKS: Conceptualization, Data curation, Formal analysis, Investigation, Methodology, Validation, Visualization, Writing – original draft, Writing – review & editing. JD: Conceptualization, Methodology, Project administration, Writing – original draft. RG: Conceptualization, Methodology, Writing – review & editing. SC: Investigation, Writing – original draft. RK: Methodology, Project administration, Resources, Supervision, Writing – review & editing. US: Conceptualization, Investigation, Methodology, Project administration, Resources, Supervision, Writing – review & editing.
